# Renewable hydrocarbons from sugarcane

**DOI:** 10.1186/1753-6561-8-S4-O35

**Published:** 2014-10-01

**Authors:** Adilson Liebsch

**Affiliations:** 1 Amyris Brasil Ltda, Campinas, São Paulo, Brazil

## 

Amyris is a renewable products company providing sustainable alternatives to a broad range of petroleum-sourced products. Amyris applies its industrial synthetic biology platform to convert plant sugars into a variety of molecules - flexible building blocks that can be used in a wide range of products.

Amyris's initial portfolio of commercial products is based on Biofene^®^, Amyris's brand of farnesene, a long-chain branched hydrocarbon, manufactured using our engineered microbes in fermentation.

First generation renewable fuels have been an important part of the effort to reduce the world's petroleum dependence and greenhouse gas emissions associated with transportation fuels. However, these first generation renewable fuels have some limitations, ranging from lower energy density than petroleum fuels and lack of fungibility with existing petroleum distribution systems.

Amyris renewable fuels are designed to be optimal transportation fuels. Specifically, our fuels are designed to be drop-in, cost competitive replacements for petroleum-derived fuels, compatible with existing engines yet with superior performance.

Building on our Biofene hydrocarbon building block, we are currently selling renewable diesel in metropolitan areas in Brazil and are pursuing industry certification for our renewable jet fuel.

